# Cannabinoid Receptor Type-2 in B Cells Is Associated with Tumor Immunity in Melanoma

**DOI:** 10.3390/cancers13081934

**Published:** 2021-04-16

**Authors:** Thomas Gruber, Steve Robatel, Mirela Kremenovic, Lukas Bäriswyl, Jürg Gertsch, Mirjam Schenk

**Affiliations:** 1 Institute of Pathology, University of Bern, 3008 Bern, Switzerland; gruber-thomas@bluewin.ch (T.G.); steve.robatel@pathology.unibe.ch (S.R.); mirela.kremenovic@pathology.unibe.ch (M.K.); lukas.baeriswyl@pathology.unibe.ch (L.B.); 2 Graduate School GCB, University of Bern, 3012 Bern, Switzerland; 3 Institute of Biochemistry and Molecular Medicine, University of Bern, 3012 Bern, Switzerland; juerg.gertsch@ibmm.unibe.ch

**Keywords:** cannabinoid receptor type-2, CB2R, endocannabinoid system, regulatory B cells, melanoma

## Abstract

**Simple Summary:**

In this study we investigated the role of cannabinoid receptor 2 (CB2R) on immune cells in melanoma and found significantly improved overall survival in patients with high intra-tumoral CB2R gene expression. In human melanoma, CB2R is predominantly expressed in B cells, as shown using a previously published single-cell RNA sequencing (scRNA-seq) dataset and by performing RNAscope. In a murine melanoma model, tumor growth was enhanced in CB2R-deficient mice. In-depth analysis of tumor-infiltrating lymphocytes using scRNA-seq showed less differentiated B cells in CB2R-deficient tumors, favoring the induction of regulatory T cells (T_reg_) and an immunosuppressive tumor microenvironment. Taken together, these data indicate a central role of CB2R on B cells in regulating tumor immunity. These results contribute to the understanding of the role of CB2R in tumor immunity and facilitate the development of new CB2R-targeted anti-cancer drugs.

**Abstract:**

Agents targeting the endocannabinoid system (ECS) have gained attention as potential cancer treatments. Given recent evidence that cannabinoid receptor 2 (CB2R) regulates lymphocyte development and inflammation, we performed studies on CB2R in the immune response against melanoma. Analysis of The Cancer Genome Atlas (TCGA) data revealed a strong positive correlation between CB2R expression and survival, as well as B cell infiltration in human melanoma. In a murine melanoma model, CB2R expression reduced the growth of melanoma as well as the B cell frequencies in the tumor microenvironment (TME), compared to CB2R-deficient mice. In depth analysis of tumor-infiltrating B cells using single-cell RNA sequencing suggested a less differentiated phenotype in tumors from *Cb2r^−/−^* mice. Thus, in this study, we demonstrate for the first time a protective, B cell-mediated role of CB2R in melanoma. This gained insight might assist in the development of novel, CB2R-targeted cancer therapies.

## 1. Introduction

Malignant melanoma is a highly aggressive form of skin cancer that constitutes only about 5% of all skin cancer cases, yet accounts for the majority (60–80%) of deaths from skin cancer [1]. Melanomas can be divided into three subtypes: mucosal melanomas, ocular melanomas and the most prevalent form, cutaneous melanomas [2]. Cutaneous melanoma (hereafter called melanoma) incidences and mortality are steadily increasing, with almost 300,000 new cases estimated worldwide in 2018 [3,4]. Tumor immunotherapy using immune checkpoint blockade (ICB) led to recent successes in melanoma treatment [5]. However, ICB fails to induce durable responses in a large proportion of cancer patients [6], and there is a huge demand for novel targets and new treatment strategies.

The endocannabinoid system (ECS) represents an emerging target in a variety of diseases, including cancer. Various cancer treatments harnessing the ECS are under pre-clinical investigation or are currently used in clinical trials [7,8,9]. The ECS consists of the endocannabinoid ligands 2-arachidonglycerol (2-AG) and *N*-arachidonoylethanolamine (anandamide) which activate the G-protein coupled receptors, cannabinoid receptor type-1 (CB1R) and cannabinoid receptor type-2 (CB2R), which were first cloned in the 1990s [10,11,12,13,14]. CB1R is primarily expressed in the central nervous system and mediates the psychoactive effects of Δ-9-tetrahydrocannabinol (Δ9-THC) and other cannabinoids [13]. The peripheral cannabinoid receptor CB2R, encoded by *CNR2*, is mainly expressed on immune cells, predominantly on B cells [14,15]. Previous studies have shown that CB2R is essential for the retention of immature B cells in bone marrow, thereby regulating B cell development [16,17]. The precise function of CB2R on immune cells of the tumor microenvironment (TME) is still elusive and correlations between the expression of CB2R and overall patient survival largely differ between cancer types [7,18]. In human metastatic melanoma, B cells constitute about one third of all tumor infiltrating immune cells and can promote or inhibit tumor growth, depending on their immune phenotype [19,20,21]. Accumulating evidence shows that naïve B cells are able to promote the differentiation of regulatory T cells (Treg), which play a key role in immune homeostasis by suppressing abnormal or excessive immune responses [22]. In tumor immunity, Treg cells are involved in tumor development and progression by inhibiting tumor-specific immune responses [23]. Given their high prevalence in tumors and their importance in tumor immunity, B cells may represent a potential target for tumor immunotherapy.

Despite recent advances in understanding the role of the ECS in cancer, comprehensive data-based mechanistic studies that enable more effective CB2R-targeted drug development are largely missing [24,25,26,27]. In this study, using data from The Cancer Genome Atlas (TCGA)’s melanoma cohort, human melanoma tissues and murine models, as well as single-cell RNA sequencing, we uncover a critical role of CB2Rs in B cells in cutaneous melanoma.

## 2. Materials and Methods

### 2.1. Tissue Culture

Murine B16F10 melanomas were purchased from American Type Culture Collection (ATCC, Wesel, Germany) and cultured in complete RPMI-1640 medium (Sigma Aldrich, Buchs, Switzerland); supplemented with 10% FBS, 100 units/mL penicillin, 100 μg/mL streptomycin, 1mM sodium pyruvate and 2 mM L-glutamine).

### 2.2. Mice, Tumor Inoculation and In Vivo Studies

We purchased the experimental C57BL/6J (B6) mice from Janvier Labs (Le Genest-Saint-Isle, France). The *Cnr2^−/−^* (B6.129P2-Cnr2tm1Dgen/J on B6 background) mice were obtained from The Jackson Laboratory. Prior to experiments, we co-housed randomized sex- and age-matched mice for at least two weeks. On day 0, tumors were engrafted by subcutaneous (s.c.) injection of 2 × 10^5^ B16F10 melanoma cells onto the left flanks of the mice. To deplete B cells in experimental animals, mice were treated with intra-peritoneal (i.p.) injection of anti-CD19 (clone 1D3) and anti-B220 (clone RA3.3A1/6.1) monoclonal antibodies (Bio X Cell) every 5 days, starting three weeks before tumor inoculation [28]. Tumor growth was followed by measuring two dimensions using a digital caliper in a blinded fashion. Tumor volume was calculated using the following formula $V=\frac{\left(length\times width^{2}\right)}{2}$ [29]. On day 14 or 15 post-tumor-inoculation or when tumor volume exceeded 1000 mm^3^, mice were euthanized and the tumors were analyzed. All mice were housed in specific pathogen-free conditions in the Central Animal Facility (CAF). All animal experiments were performed in accordance with federal regulations and approved by the Cantonal Veterinary Office (BE70/19).

### 2.3. Flow Cytometric Analyses and Cell Sorting

To analyze their cellular composition by means of fluorescence-activated cell sorting (FACS), murine tumors were processed as previously described [30]. Briefly, the tumors were removed and mechanically dissociated and filtered twice through a 40-μM strainer (ThermoFisher, Waltham, MA, USA) to obtain a single-cell suspension. Fc-receptors were blocked using an anti-mouse CD16/32 antibody (2.4G2, generated in house) for 15 min. To exclude dead cells from further analysis we used the Zombie Aqua or UV™ Fixable Viability Kit (Biolegend, San Diego, CA, USA). Subsequently, cells were incubated with cell surface marker-specific antibodies in FACS buffer (PBS with 2% FBS and 1 mM EDTA) for 45 min on ice. The following antibodies targeting mouse surface antigens were used: anti-CD45.2 (104), anti-CD3 (145-2C11), anti-CD4 (RM4-5), anti-CD8 (53-6.7), anti-CD19 (6D5), anti-NK1.1 (PK136), anti-CD11b (M1/70) and anti-Gr-1 (RB6-8C5). Intracellular antibody labeling was performed using the eBioscience™ Foxp3/Transcription Factor Staining Buffer Set following the manufacturer’s instructions. The following antibodies were used: anti-Ki67 (16A8), anti-IFNγ (XMG1.2), anti-IL-10 (JES5-16E3) and anti-Foxp3 (MF-14); all antibodies were purchased from Biolegend. The samples were acquired using a Beckman Coulter CytoFLEX S flow cytometer and the data were analyzed using Flowjo (Treestar, BD, Eysins, Switzerland). For single-cell RNA sequencing, CD19^+^ B cells were FACS purified using a Moflo Astrios EQ cell sorter (Beckman Coulter, Brea, CA, USA) and processed immediately.

### 2.4. Fluorescence In Situ Hybridization (FISH) Using RNAscope

Human melanoma formalin-fixed paraffin-embedded (FFPE) biopsies were obtained and analyzed in accordance with the guidelines of the Cantonal Ethics Committee (KEK) in Bern under approved protocols (KEK ID: 2017-02246). For the in situ detection of *CD19* (RNAscope^®^ Probe -Hs-CD19-C2) and *CNR2* (RNAscope^®^ Probe -Hs-CNR2), the RNAscope^®^ Multiplex Fluorescent Assay v2 kit was used according to the manufacturer’s protocols (ACD, Biotechne, MN, USA). Whole slide images were acquired using a Pannoramic 250 Flash II (3D Histech) and analyzed using QuPath [31].

### 2.5. Single-Cell RNA Sequencing

FACS-purified B cells from two mice per group were pooled and analyzed using the 10X Chromium System. Library preparation was done according the 10X Genomics protocols (Chromium Single-cell 3′ GEM Library and Gel Bead Kit v3) and sequenced on NovaSeq 6000 (Illumina SP flow cell, 100 bp paired-end reads). FASTQ files were generated using bcl2fastq v2.20.0.422 from Illumina. Using the default settings in cellranger 3.1.0, reads were aligned to the mouse genome mm10 from ENSEMBL GRCm38 and all four runs from the same sample were aggregated. This resulted in 4568 wt and 5273 *Cnr2*^−/−^ B cells. Calculations were performed on UBELIX (http://www.id.unibe.ch/hpc, accessed on 4 October 2019), the HPC cluster at the University of Bern. Further QC and downstream analysis was performed using Seurat in R, as previously reported [32]. Cells that contained a percentage of mitochondrial transcripts >15% were filtered out. To remove putative empty droplets, cells with total molecule counts of <1500 and features of <750, were additionally filtered out, resulting in 3187 wt and 3194 *Cnr2*^−/−^ B cells that passed QC metrics, with a median of 3474 and 3409 counts/cell, respectively. Normalization was performed using the “LogNormalize” method with a scale factor of 10,000. Samples were integrated using default settings. Gene ontology (GO) term enrichment analyses between clusters were performed using the clusterProfiler package in R [33].

### 2.6. TCGA Data Collection and Analysis

We used the skin cutaneous melanoma (SKCM) cohort from The Cancer Genome Atlas (TCGA) and downloaded the gene expression data, as well as clinical information, using GDCRNATools [34]. Normalization of counts was achieved using the default parameters of the DESeq2 package in R [35]. Lasso regression to *CNR2* was performed using the glmnet package in R [36]. The Human Gene Atlas library in Enrichr was used for gene set enrichment of selected variables [37]. Kaplan–Meier survival analyses were performed by stratifying the samples into *CNR2* high and low expressing groups using the top and bottom quartiles and these were analyzed using the survminer package in R (http://www.sthda.com/english/rpkgs/survminer/, accessed on 7 November 2019). The survival time was calculated by subtracting the days from initial diagnosis to diagnosis of the tumor used for RNA sequencing (“days_to_submitted_specimen_dx” taken from http://firebrowse.org/?cohort=SKCM, accessed on 3 November 2019, file “Merge_Clinical (MD5)”) from the overall survival (OS) time.

### 2.7. Analysis of Gene Expression Omnibus (GEO) Dataset

We obtained previously published single-cell RNA-sequencing data on CD45^+^ and CD45^−^ FACS purified cells from human melanoma samples from GSE72056 [38]. The analysis was performed as previously described [30].

### 2.8. Statistical Analysis

GraphPad Prism version 7.0 or higher (GraphPad Software version 7.0) or R was used for the statistical analyses. Statistical significance was determined as described in each Figure legend. Each dot represents an individual mouse or a single cell for the scRNA-seq data. All bar graphs show means and SEM values. * *p* ≤ 0.05; ** *p* ≤ 0.01; *** *p* ≤ 0.001; **** *p* ≤ 0.0001.

## 3. Results

### 3.1. B Cell-Associated CB2Rs Play a Role in Human Melanoma Progression

**Figure 1:**
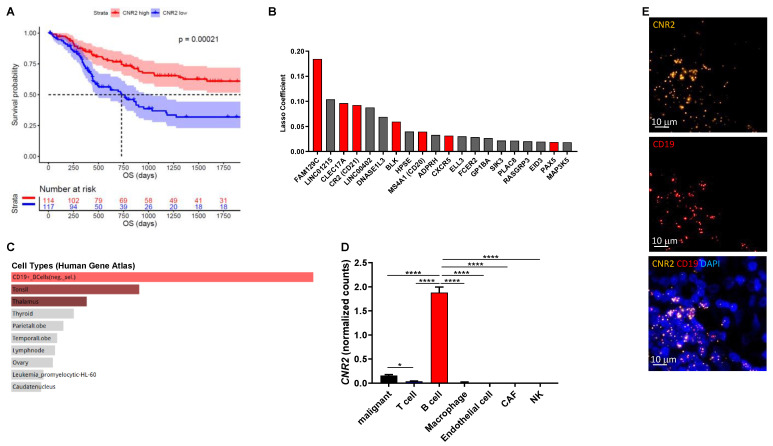
*CNR2* is positively associated with increased overall survival (OS) in human melanoma patients and is primarily expressed in B cells. (**A**) Survival curves of melanoma patients with high vs. low *CNR2* expression (top vs. bottom quartile) up to 5 years OS; log-rank test. Median survival for *CNR2* high (*n* = 114) and low (*n* = 117) was 3818 and 730 days, respectively. (**B**) Top 20 genes predictive for *CNR2* expression in melanoma as determined by lasso regression. Red bars represent B-cell-related genes; *n* = 472. (**C**) Gene set enrichment analysis of genes with lasso coefficient > 0 (*n* = 73) using Human Gene Atlas library. (**D**) *CNR2* expression in various cell types as determined by single-cell RNA sequencing; Kruskal–Wallis test followed by Dunn’s multiple comparisons testing. Bar graphs are shown as mean +/− SEM. * *p* ≤ 0.05; **** *p* ≤ 0.0001. (**E**) Representative RNAscope-derived immunofluorescence images on human melanoma tissue sections.

Cannabinoids that selectively target CB1Rs or CB2Rs have potential for the treatment of various forms of tissue injury and inflammatory diseases [39]. Although selectively targeting CB2Rs has been shown to attenuate tumor growth in mice [40,41], little is known about their mechanism of action in cancer, especially related to tumor immunobiology. In this study we investigated the role of CB2R on immune cells in melanoma. Initially, we analyzed gene expression data from the TCGA cohort (*n* = 472) and found significantly improved overall survival in patients with high intra-tumoral CNR2 (CB2R) gene expression (Figure 1A) but not CNR1 (CB1R). Genes predictive for CNR2 expression were selected using machine learning-based regression by means of the lasso method (Figure 1B). These genes were primarily expressed in B cells, as determined by gene set enrichment analysis using the Human Gene Atlas library in Enrichr (Figure 1C) [42]. We confirmed the predominant expression of CB2R in B cells using a previously published single-cell RNA sequencing dataset (Figure 1D) and by performing RNAscope for CD19 and CNR2 on human melanoma tissues (Figure 1E). Of note, only few malignant cells expressed detectable levels of CNR2. Taken together, these data indicate a central role of CB2Rs on B cells in regulating tumor immunity.

### 3.2. Cnr2 Deficiency Leads to Enhanced Melanoma Growth and B Cell Infiltration in Murine Melanoma

**Figure 2:**
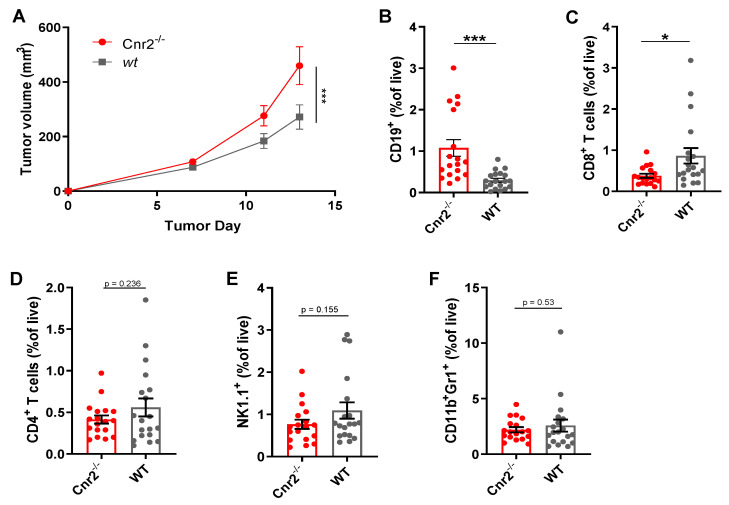
CB2R regulates tumor growth and B cell infiltration in murine melanoma. (**A**) Tumor growth shown as mean ± SEM. Statistical significance was determined by two-way ANOVA followed by Šidák’s multiple comparisons test, *n* = 10. (**B**–**F**) Frequencies of indicated immune cell types at day 14 post-tumor inoculation as determined by flow cytometry, gated on live CD45^+^ cells. *n* = 17–18; two-tailed, unpaired Student’s *t*-test. * *p* ≤ 0.05; *** *p* ≤ 0.001.

To gain a better mechanistic insight into the role of CB2Rs in melanoma, we next established the murine B16F10 cutaneous melanoma model in *Cnr2*^−/−^ and wild-type (*wt*) mice. Melanoma growth was significantly enhanced in CB2R-deficient mice (Figure 2A). Next, we quantified the immune cell infiltration in tumors from *Cnr2*^−/−^ and wt mice using flow cytometry. The most significant differences we observed were an increase in the B cell frequencies in CB2R-deficient mice (Figure 2B), together with reduced frequencies of CD8^+^ T cells (Figure 2C). However, no quantitative difference in CD4^+^ T cells, NK cells and myeloid-derived suppressor cells was observed (Figure 2D–F).

### 3.3. Tumor-Infiltrating B Cells of Cnr2-Defficient Mice Display Signs of Impaired Differentiation and Catabolism

**Figure 3:**
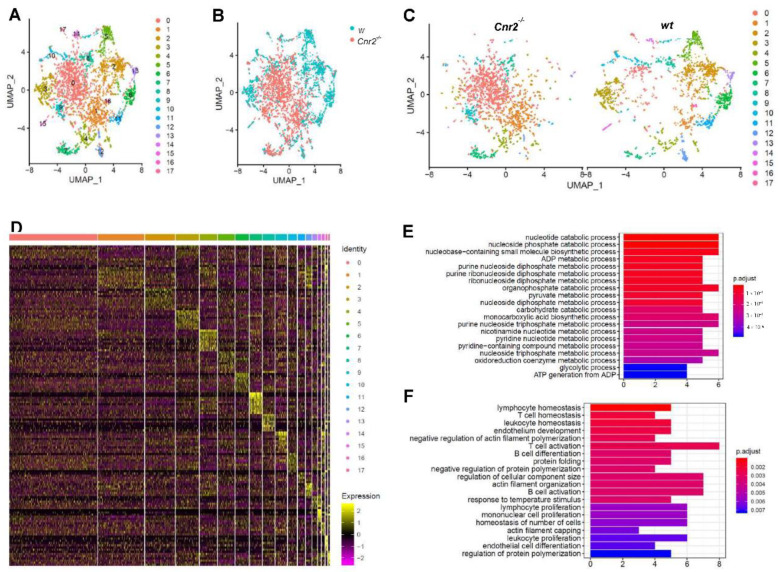
Tumor-infiltrating B cells from *Cnr2^−/−^* mice have reduced metabolic activity, along with impaired differentiation and activation. Tumor-infiltrating B cells from wt and *Cnr2^−/−^* mice were purified using flow cytometry at day 14 post-tumor inoculation and analyzed by means of scRNA-seq. (**A**–**C**) UMAP dimensionality reduction of 6381 combined B cells. (**D**) Heatmap showing the top 10 upregulated genes (highest fold change) for each cluster. (**E**) Gene ontology analysis for biological processes of downregulated genes with log fold change threshold of 0.25 and minimum prevalence of 0.2 from cluster 0 (*n* = 37) and (**F**) cluster 1 (*n* = 56).

For an in-depth characterization of tumor-infiltrating B cells in *Cnr2*^−/−^ and wt mice, we FACS-purified CD19^+^ cells and performed single-cell RNA sequencing. For subsequent analysis, we employed dimensionality reduction using Uniform Manifold Approximation and Projection (UMAP), which is superior to t-Distributed Stochastic Neighbor Embedding (tSNE) in preserving the global structure of the data [43]. Thereby, we identified 17 different clusters of B cells (Figure 3A). Remarkably, B cells isolated from *Cnr2*^−/−^ mice were strongly separated from those isolated from wt mice (Figure 3B). CB2R-deficient B cells were mainly located at the center in clusters 1 and 2, surrounded by distinct B cell clusters from wt mice (Figure 3C). This indicates an earlier development stage of CB2R-deficient B cells compared to the more differentiated B cells from wt mice. The biggest *Cnr2*^−/−^ clusters (cluster 1 and 2) showed only a few significantly upregulated genes (*n* = 5 and 22), whereas more genes were downregulated (*n* = 37 and 56) (Figure 3D). Gene ontology term enrichment analysis of the downregulated genes in B cells of *Cnr2*^−/−^ mice showed impairments in catabolic processes in cluster 0 (Figure 3E) and deficient B cell differentiation and activation in cluster 1 (Figure 3F).

### 3.4. Cnr2-Deficient B Cells Drive the Induction of Treg

**Figure 4:**
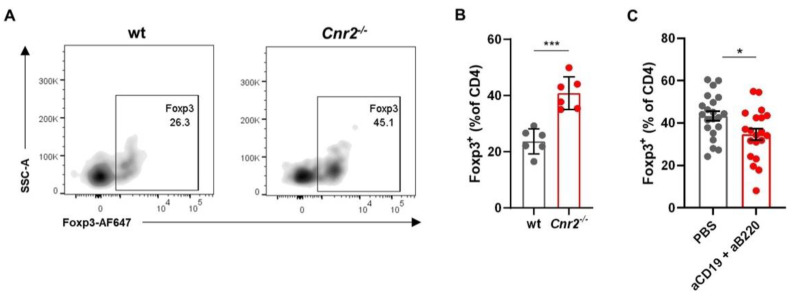
CB2R mediates tumor growth control by regulating Breg infiltration and Treg induction. Tumors from wt and *Cnr2^−/−^* mice were isolated at day 14 post-tumor inoculation and analyzed by means of flow cytometry. (**A**) Representative flow cytometry plots and (**B**) frequencies of intratumoral Foxp3^+^ cells as percentages of CD4^+^ T cells in wt and *Cnr2^−/−^* mice (*n* = 6). (**C**) Intratumoral Foxp3^+^ cell frequencies in *Cnr2^−/−^* mice with or without B cell depletion using anti-CD197 and anti-B220 monoclonal antibodies (mAbs). Statistical analyses were performed using two-tailed, unpaired Student’s *t*-tests. Error bars show mean ± SEM. * *p* ≤ 0.05; *** *p* ≤ 0.001.

Increased tumor growth in CB2R-deficient mice, associated with the enhanced presence of largely undifferentiated B cells indicates a regulatory phenotype of these B cells. One immunosuppressive function of Breg is the induction of Treg [44]. The Breg phenotype of *Cnr2^−/−^* B cells is supported by our flow cytometric analysis, which shows strongly elevated frequencies of Foxp3^+^ Treg in CB2R-deficient mice compared to wt mice (Figure 4A,B). Upon B cell depletion using anti-CD19 and anti-B220 depleting antibodies, Treg frequencies were reduced (Figure 4C, Supplemental Figure S1), suggesting that CB2R regulates tumor immunity by reducing Breg-mediated Treg induction.

## 4. Discussion

Different cannabinoids that target CBRs have been studied as potential cancer treatments in pre-clinical cancer models and clinical trials [7,8]. Most of the knowledge about CB2R in melanomas is derived from murine studies, whereas studies examining CB2R in human melanomas remain sparse. Using the B16F10 murine cutaneous melanoma model, a remarkable reduction in tumor growth upon treatment with the selective CB2R agonists GW833972A and JWH-133 was found [45]. Moreover, using immunohistochemistry it has been shown that CB2R is upregulated in human melanomas compared to normal tissue [46], but the origin of CB2R expression was not elucidated. In this study, we have demonstrated for the first time a protective role of CB2R expressed in B cells in human cutaneous melanoma. Therefore, CB2R represents a potential prognostic biomarker and thus requires further prospective studies in this regard.

Our findings show a strong positive correlation between CB2R expression and overall survival in cutaneous melanoma, concurrent with an increased infiltration of intratumoral B cells. Furthermore, we observed enhanced B16 melanoma growth in mice that were deficient in CB2R. Previous reports have demonstrated that CB2Rs are essential for the retention of immature B cells in the bone marrow and thus regulate their development [16,17]. Therefore, CB2R deficiency leads to impaired B cell development and differentiation, inducing a release of immature B cells into the circulation [16,17,24,47]. In line with these results, we found a striking elevation of B cell infiltration in the TME of *Cnr2^−/−^* mice. Indeed, our single-cell RNA-sequencing analysis of tumor infiltrating B cells revealed an impairment of B cell differentiation and activation, as well as metabolic alterations in *Cnr2^−/−^* mice, suggesting that immature B cells released from the bone marrow infiltrated the tumor. The immunosuppressive phenotype of these B cells is indicated by our finding that the frequencies of CD4^+^Foxp3^+^ regulatory T cells (Tregs) were increased in *Cnr2*^−/−^ mice and reduced upon B cell depletion. Furthermore, the TME of *Cnr2^−/−^* mice displayed significantly reduced frequencies of CD8^+^ T cells. Based on these findings, we hypothesize that CB2R deficiency triggers the infiltration of immature B cells and the induction of Treg, thereby dampening the tumor-specific immune response. We are further investigating these altered B cell subsets from *Cnr2*^−/−^ mice to provide key biological insights on the pathways through which CB2R modulates B cell differentiation and cancer. Thus, in this report, we demonstrate for the first time a B-cell-mediated protective role of CB2R against cutaneous melanoma. These findings provide further understanding of the complex biology of CB2R in the immune system and warrant further investigation. Moreover, they support the development of drugs targeted at this receptor as potential cancer treatments.

## 5. Conclusions

Taken together, our study provides additional evidence that CB2Rs expressed in B cells are regulators of tumor immunity and exhibit a protective association in humans. This insight might help to conceive and develop novel CB2R-targeted cancer therapies.

## Data Availability

The data presented in this study are available on request from the corresponding author. The data are not publicly available due to privacy.

## Supplementary Materials

The following are available online at https://www.mdpi.com/article/10.3390/cancers13081934/s1, Figure S1: CD19^+^ B cell frequencies in indicated tissues with or without B cell depletion. B cells were depleted using anti-CD19 and anti-B220 depleting antibodies and analyzed at day 14 post tumor inoculation by flow cytometry (*n* = 6–7). Statistical analyses were performed using two-tailed, unpaired student’s *t*-tests. Error bars show mean ± SEM. * *p* ≤ 0.05; **** *p* ≤ 0.0001.
