# Supplementary material for: Cannabinoid Receptor Type-2 in B Cells Is Associated with Tumor Immunity in Melanoma
Source: Cancers (Basel). 2021 Apr 16;13(8):1934. doi: 10.3390/cancers13081934 (PMC8073134; doi:10.3390/cancers13081934)
Supplement: Supplementary file 1 [file cancers-13-01934-s001.zip › cancers-1170562-supplementary.pdf]

cancers

Article

Cannabinoid receptor type-2 in B cells is associated with tumor immunity in melanoma

Supplementary material

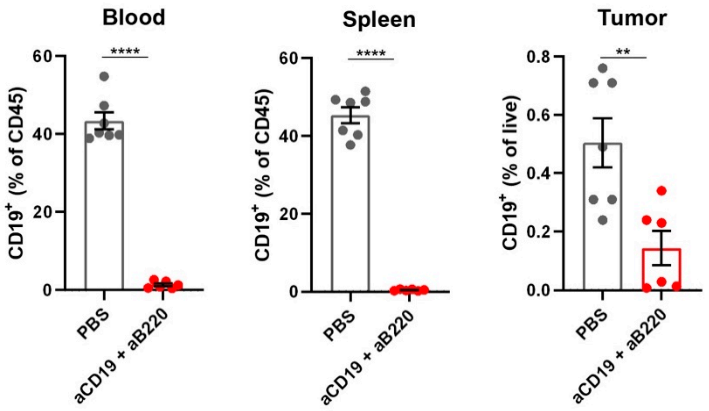

Figure S1. CD19⁺ B cell frequencies in indicated tissues with or without B cell depletion. B cells were depleted using anti-CD19 and anti-B220 depleting antibodies and analyzed at day 14 post tumor inoculation by flow cytometry (n = 6-7). Statistical analyses were performed using two-tailed, unpaired student's *t*-tests. Error bars show mean ± SEM. * $p \leq 0.05$; **** $p \leq 0.0001$.
